# Process evaluation of discharge planning implementation in healthcare using normalization process theory

**DOI:** 10.1186/s12911-016-0285-4

**Published:** 2016-04-27

**Authors:** Sofi Nordmark, Karin Zingmark, Inger Lindberg

**Affiliations:** Division of Nursing, Department of Health Science, Luleå University of Technology, Luleå, Sweden; Department of Healthcare Administration, Norrbotten County Council, Luleå, Sweden; Department of Research and Development, Norrbotten County Council, Luleå, Sweden

**Keywords:** Discharge planning process, Implementation, Normalization Process Theory, Qualitative research

## Abstract

**Background:**

Discharge planning is a care process that aims to secure the transfer of care for the patient at transition from home to the hospital and back home. Information exchange and collaboration between care providers are essential, but deficits are common. A wide range of initiatives to improve the discharge planning process have been developed and implemented for the past three decades. However, there are still high rates of reported medical errors and adverse events related to failures in the discharge planning. Using theoretical frameworks such as Normalization Process Theory (NPT) can support evaluations of complex interventions and processes in healthcare. The aim of this study was to explore the embedding and integration of the DPP from the perspective of registered nurses, district nurses and homecare organizers.

**Methods:**

The study design was explorative, using the NPT as a framework to explore the embedding and integration of the DPP. Data consisted of written documentation from; workshops with staff, registered adverse events and system failures, web based survey and individual interviews with staff.

**Results:**

Using the NPT as a framework to explore the embedding and integration of discharge planning after 10 years in use showed that the staff had reached a consensus of opinion of what the process was (coherence) and how they evaluated the process (reflexive monitoring). However, they had not reached a consensus of opinion of who performed the process (cognitive participation) and how it was performed (collective action). This could be interpreted as the process had not become normalized in daily practice.

**Conclusion:**

The result shows necessity to observe the implementation of old practices to better understand the needs of new ones before developing and implementing new practices or supportive tools within healthcare to reach the aim of development and to accomplish sustainable implementation. The NPT offers a generalizable framework for analysis, which can explain and shape the implementation process of old practices, before further development of new practices or supportive tools.

## Background

Discharge planning (DP) is a complex process that aims to secure the patients’ care transition from home to the hospital and back home [1]. Patients’ needs and resources are identified, and multidisciplinary interventions from different care providers are coordinated to match the identified needs. Deficits in the discharge planning process (DPP), such as poor communication and collaboration between care providers, can cause serious breakdowns in the continuity of care [2, 3] that can lead to consequences for the patient, such as delayed discharge, readmission [4, 5] and inadequate post-discharge care [6].

Discharge planning as a concept has been described in the literature for the past three decades [1, 7], and even if the process definition diverse there are common threads [8]. Across many Western countries, the main structure of the process is similarly described, and there are similar launched guidelines and strategies for good practice in the DPP. In Australia, there are strategies to improve patient discharge by rewarding hospitals with good practice [9]. In Sweden, there are laws and guidelines that regulate the coordination of information and collaboration from the patient’s hospital admission to discharge [10, 11]. In the UK, the National Health Service states that every NHS patient should have a discharge plan starting from admission [12]. In Ireland, the National Health Strategy, Quality and Fairness states that patients’ hospital discharge is a process and not an isolated event and should therefore start at admission [13]. In the US, DP not only is a basic hospital function but is also legally mandated [14]. A discharge plan must be developed by a registered nurse (RN), a social worker or other appropriately qualified personnel, and it should be initiated as soon as possible after admission. Common in these countries’ guidelines is that the decision that the patient is medically fit for discharge can be made only by the patient’s consultant or another physician responsible for the patient’s care but, the DP is performed by a RN, a social worker or a specific discharge planner responsible for coordinating the patient’s discharge [9–14]. Furthermore these guidelines also state that at a patient’s discharge, some type of discharge summary must be sent from the hospital to the general practitioner/ primary healthcare provider.

The literature reveals that deficits in communication and collaboration during the DPP are common [15]. Among the mentioned barriers that impede timely and secure DP are high workloads among RNs [8, 16], variable schedules among healthcare personnel [17, 18], a lack of trained personnel [19, 20], and ineffective communication for timely exchange [21, 22]. Unclear roles [23], unclear routines, and unstructured information [24] also impede the DP, as does a changing healthcare system, which has seen a reduced number of hospital beds and shorter hospital stays [25]. An increasing elderly population and an increasing amount of people with chronic illness leads to greater pressure on healthcare. These demographic and social changes demand good planning, coordination, and a timely exchange of patient-related information during the DPP to avoid failures. The time available for discharge preparation has been significantly reduced [26]. A wide range of initiatives to improve the DP have been created, including; education and training for personnel in DP [27, 28], specification of roles for people performing the DP e.g., the discharge planner [29], specific discharge screening tools and models [30, 31], standardized discharge letters [32], and medication reconciliation [23]. Implementations of information and communication technology (ICT) solutions to support the information exchange are also common [33]. However, ICT solutions, such as electronic health records (EHR) are not necessary designed to support DPP in particular. There are still high rates of reported medical errors and adverse events related to failures in the DPP [15, 34], even though the effectiveness of DP interventions are often measured by mortality rate, patient satisfaction, costs, and unplanned readmissions.

Implementing and evaluating interventions such as new technologies and processes in healthcare is complex and demanding. Using theoretical frameworks such as Normalization Process Theory (NPT) can support evaluations of complex interventions and processes in healthcare [35]. NPT explains how material practices become routinely embedded in their contexts by referencing four generative mechanisms (coherence, cognitive participation, collective action, and reflexive monitoring), and by focusing on the work that people perform. It explains how the work, individual and collective, of implementing new practices requires continuous investment in plans of action that carry forward in time and space. The starting point of NPT is coherence, or the sense-making work people perform individually and collectively. An important element is for a group to collectively understand how a practice is different from others and to understand the aims and expected benefits of this practice. However, it is also important for individuals to understand their specific responsibilities. According to NPT, cognitive participation is the relational work that people perform to build and sustain a community of practice around a new intervention. It is closely bound to the norms and conventions within the social matrices where actors find themselves working. Collective action refers to the effect a new practice has on interactions and relations. It also refers to the fit between the new practice and existing skill sets and the overall organizational context. Reflexive monitoring is the appraisal work people perform to assess and understand how a new practice affects them and others around them.

Deficits in the DPP have been well studied, and so have initiatives to improve the DPP. However, most studies have focused on what barriers there are, and how they can be overbridged. Fewer studies have focused on personnel’s embedding and integration of the DPP. It is important to gain knowledge of how embedded and integrated the process is to better target initiatives for improvement.

### Aim

The aim of this study was to explore the embedding and integration of the DPP from the perspective of registered nurses (RNs), district nurses (DNs) and homecare organizers (HCOs).

## Methods

This study was designed as an explorative study using the NPT as a framework to explore [35, 36] the embedding and integration of the DPP. Separate analyses of data from different sources (individual interviews with DNs and HCOs, survey with RNs and DNs) had been performed previously, and the results indicated that there were several similarities in the descriptions of obstacles and feasibilities connected to the DPP. To reach a better understanding of the entire process, qualitative data from these sources were therefore brought together with unanalysed data from other sources (individual interviews with RNs, workshops, registered adverse events and registered system failures) and analysed using NPT as a framework.

### Context

This study was part of a larger project called Future’s Innovative Work Practices in Healthcare and Welfare (FIA), conducted between 2009 and 2012 in a county in northern Sweden [37]. The purpose of the project was to create work methods and ICT solutions that increased the accessibility, safety, quality, and efficiency of healthcare, lowering costs and creating regional growth. The project had an agile work method [38, 39]. It was considered to be of significance to include RNs from the central county hospital, DNs from primary healthcare, and HCOs from the community as partners throughout the entire project process. RNs, DNs and HCOs were involved as end-users from the early stages of the project to advocate their specific needs. The project resulted in five ICT solutions aiming to support the DPP; an electronic shared calendar, videoconference as a way of meeting for DPC, development of the electronic information system offering an attached file with the patient’s status in the request for DPC, follow-up of the agreed discharge plan, and a surveillance list to keep track of discharged patients’ in need of follow-up. This study focused on exploring the embedding and integration of DPP from the perspective of RNs, DNs and HCOs, using the NPT as a framework, before new ICT solutions were developed and tested.

**Fig. 1:**
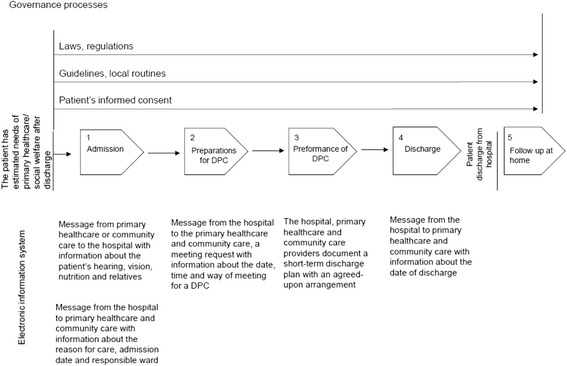
Overview of the discharge planning process and the information exchange through the electronic information system. Shows the discharge planning process with its steering processes and sub processes. It also describes when in the process messages are sent between care providers in the county electronic information system, and type of information in each message

In the county where the project took place, the DPP was first implemented in 1992. The patient-related information exchange during the DPP was initially performed by phone or by facsimile because the county council and the municipalities had different electronic medical records. In 2002, local guidelines for the performance of DPP were agreed upon by the county council and the county municipalities, and a specific electronic information system was developed and implemented to secure the information exchange between the hospital, primary healthcare and community care during the DPP. The communication tool had a structure that allowed free text messages to be sent at patient’s admission to the hospital, before DPC, after the DPC and at the patient’s discharge, Fig. 1. Thus, much of the patient-related information had to be doubly documented, first in the medical record system for internal use and then in the electronic information system for external use.

From 1992 to 2012, the physician at the hospital ward was responsible for the DP, according to the law [10] and the regulation [11]. However, it was the RN at the hospital who performed the DP, from admission to discharge. The DN was employed by the county council and responsible for coordinating and performing home healthcare. The HCO was employed by the municipality and responsible for coordinating social care. The RN, DN, and HCO were central in the performance of the DPP.

### Participants and procedure

**Fig. 2:**
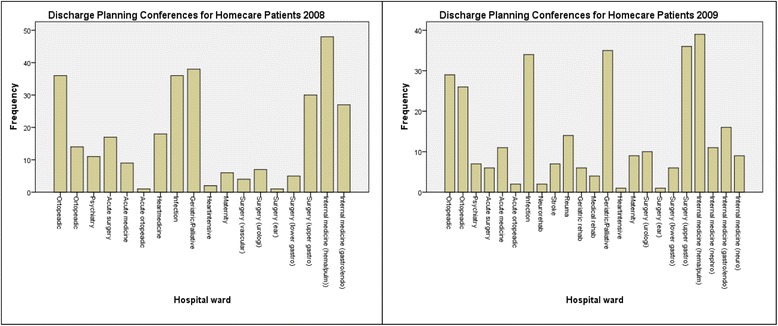
Frequency of performed discharge planning conferences between project volunteer healthcare centres and the central county hospital. Shows the frequency of performed discharge planning conferences between the volunteering healthcare centre and each ward at the county hospital for 2008 and 2009. It shows most frequent discharge planning conferences was towards the orthopedic ward, infection ward, geriatric/palliative ward, one surgery ward and the internal medicine ward with pulmonary and hematological diseases

Data of all performed DPs at the care settings in a predefined area were analysed using descriptive statistics [40] to form a clear picture of the frequency of DPs. Statistics on the frequency (Fig. 2) of DPs performed between one volunteer primary healthcare centre and each ward at the central county hospital over the previous 2 years was reviewed. Five hospital wards with the highest frequency of DPs were identified: geriatric/palliative, infection, surgical, orthopaedic and pulmonary medicine/endocrinology- gastrology. Comparing statistics for these five wards helped identify the five primary healthcare centres with the most frequent discharge planning for the central county hospital. One was the volunteer primary healthcare centre, located in a smaller town with patients spread over a large geographic area. To acquire different views and information on a variety of experiences, another two of the five primary healthcare centres were selected according to where they were situated; one was located in a larger city that was close to the hospital, and one was located in the countryside. Two municipalities were also selected since they served patients in the same care catchment area as the three selected healthcare centres.

### Workshops

Informational meetings with RNs, DNs and their management were held by the first author at each care setting. Information about the project and the study was provided both verbally and in written form [41]. The same information was given to the HCOs and their management but at different occasions due to logistical problems. RNs, DNs and HCOs working with DP were invited to participate in the project. Twelve RNs agreed to participate, two from each ward except at the medical ward, where two RNs from each sub-specialty agreed to participate. Nine DNs, three from each primary healthcare centre, and five HCOs, two from one municipality and three from the other one, also agreed to participate.

### Survey

The study sample covered an entire population in a defined geographic area [42]. All RNs from the central county hospital and all DNs working with home-nursing patients in the 13 primary healthcare centres in the surrounding area were invited to participate. Those who had a temporary position and/or worked only at night were excluded. After approval from the head of each clinic, a list of potential participants from each unit was obtained with assistance from the Department of Human Resources. In total, 261 information letters (RN, *n* = 194; DN, *n* = 67) that contained an invitation to participate in the study, and the questionnaire were sent out via the county council’s internal e-mail system. By answering the web-based questionnaire, the participants gave their informed consent [41].

### Interviews

I. RNs working with DP at five different hospital wards at the central county hospital were asked to participate. They were contacted through their managers, who gave the RNs written information about the study along with a responding address form [41]. In total, twelve RNs agreed to participate (two from each hospital ward except at one ward, where two RNs from each sub-specialty agreed). They sent the responding form to the first author, who contacted them by phone to arrange a time and place to conduct individual interviews.

II. DNs working with home-nursing patients at three different primary healthcare centres, and HCOs working for two different municipalities were asked to participate. They were contacted through their managers, who gave them written information about the study along with a responding address form [41]. In total, nine DNs (three from each primary healthcare centre), and five HCOs (two from one municipality and three from the other) agreed to participate. They sent the responding form to the first author, who contacted them by phone to arrange a time and place to conduct individual interviews.

### Data collection

Using the NPT to explore the embedding and integration [35] of the DPP, the data collection was performed from documentation written from 2009 to 2010 from the following sources;

### Workshops

Written results of three workshops held in 2009 with a mixed group of twelve RNs, nine DNs and five HCOs. The workshops aimed to map the DPP with its steering processes, sub-processes, activities and roles along with hindrances and feasibilities. The workshops were held by the first author and a technical project leader. Each session lasted 60–90 min.

### Registered adverse events

A written list of adverse events in the area of DP, registered by healthcare professionals in the county councils electronic deviation-handling system from January 2009–March 2010. The list was printed from the system by the head of the department of primary healthcare who also had a role as a co-ordinator between the hospital, primary healthcare and community care concerning DP on the management level.

### Registered system failures

A list of frequently occurring problems with and failures in the electronic information system reported by end-users and registered in the county council’s electronic fault-reporting system in 2009 and 2010.

### Survey

Data were also collected from the qualitative results of a web-based survey with RNs and DNs performed in 2010. This survey was originally undertaken to explore the information exchange during the DPP. Focus of the survey was on what, when, and how information is exchanged in the process, and on the hindrances and feasibilities for the exchange. A total of 171 questionnaires were completed and returned. The response rate was 65.5 %. The survey is published in detail elsewhere [43].

### Interviews

I. Data were collected from semi-structured individual interviews with RNs performed in 2010 that aimed to explore their experiences with and views of factors that promote or inhibit the DPP. An interview guide, based on the law about communities’ responsibility to pay for certain healthcare [10] and the regulation about collaboration at hospital admission and discharge [11], was used along with six vignettes illustrating a map of the DPP with sub-process and activities. The interviews were performed by the first author and took place in a room disconnected from the workplace where the RNs worked. Interviews lasted 90 to 120 min and were tape-recorded and transcribed verbatim by the first author.

II. Data were collected from semi-structured individual interviews that took place in 2010 with DNs and HCOs. These interviews were originally undertaken to explore DNs’ and HCOs’ experiences with and views of the workflow during the DPP. The interviews focused on five areas of interest: workflow, routines, organization, documentation and expectations and demands. The interviews were performed by the first author, lasted 90 to 120 min, and took place in a room disconnected from the DNs’ and HCOs’ workplace. The interviews were tape-recorded and transcribed verbatim by the first author. The study is published in detail elsewhere [44].

### Data analysis

In this study, the NPT was used as a framework to explore the embedding and integration of the DPP with its electronic information system among RNs, DNs and HCOs after 10 years in use to understand how the DPP had operated (35). A framework analysis involves five steps: familiarizing, identifying a thematic framework, indexing, charting, and mapping and interpreting [36]. The analysis started with a close reading of the written texts from each source to become familiar with the data and to gain a sense of the whole. The NPTs’ four core constructs were discussed by the three authors; key issues and questions relevant to the study context for each one of the core areas were identified and clarified. Data that related to the four predefined core areas in the NPT; coherence, cognitive participation, collective action and reflexive monitoring were extracted from the texts by the first author. The data were coded and sorted into the four groups by moving back and forth between the text and the NPT literature [35, 45]. To support coding and sorting, Microsoft Excel 2010 was used. The various text units in each core areas were compared and discussed based on differences and similarities by the three authors. Similar text units were brought together in categories under each core area, which allowed for a comparison of core areas and categories to see if any further changes or merging was required. By mapping the data from the project work and research work onto the NPT’s (Table 1) four core areas, we furthered our knowledge and understanding of how normalized the DPP had become in daily practice. In relation to the embedding and integration of the DPP, the four core areas of NPT were defined as the following:

**Table 1:** Exploring embedding and integration of DPP using NPT for analysis of project and research results. Gives an overview of the number of text units from each data source that was sorted into the four core areas of the NPT: coherence, cognitive participation, collective action and reflexive monitoring

	Core areas			
	Coherence	Cognitive participation	Collective action	Reflexive monitoring
	What is the process?	Who performs the process?	How does the process get performed?	How is the process understood?
	How RNs, DNs and HCOs perceived the DPP and whether they experienced the DPP as valuable to them and agreed about its usefulness and purpose.	Whether RNs, DNs and HCOs saw the DPP as a legitimate part of their work and whether they supported it over time	How the DPP was provided within the existing context, how the embedding and integration work had proceeded due to knowledge and resources	How RNs, DNs and HCOs individually and collectively evaluated the DPP and its supportive tools.
	Factors that promote or inhibit the routine embedding of DPP	Factors that promote or inhibit participation in DPP	Factors that promote or inhibit enacting DPP	Factors that promote or inhibit appraisal of DPP
Data source	No. of text units			
Survey	0	1	12	0
Interview RNs	0	119	225	78
Interview DNs, HCOs	0	122	80	59
Adverse events/ information system failures	0	3	2	0
Workshops	12	8	37	6

Coherence of the DPP (What is the process?): how RNs, DNs and HCOs perceived the DPP and whether they experienced the DPP as valuable to them and agreed about its usefulness and purpose.

Cognitive participation (Who performs the process?): whether RNs, DNs and HCOs saw the DPP as a legitimate part of their work and whether they supported it over time.

Collective action (How is the process performed?): how the DPP was provided within the existing context, how the embedding and integration had proceeded, and what factors promoted or inhibited the work.

Reflexive monitoring (How is the process understood?): how RNs, DNs and HCOs individually and collectively evaluated the DPP.

### Ethical considerations

The study was approved by the Regional Ethical Review Board in Umeå, Sweden. (Dnr 09–216 M). The manager of the health care centres and social care gave permission for the studies. Both oral and written information about the studies was given. The participants were reassured that their participation was voluntary and that they could withdraw from the study at any time. To guarantee confidentiality, no individual characteristics were disclosed. Written informed consent to participate in the workshops and interview studies was obtained.

## Results

### Embedding and integration of the DPP

The results of using the NPT as a framework for exploring the embedding and integration of the DPP showed that RNs, DNs and HCOs had reached a consensus of opinion of what the process was (coherence) and how they evaluated the process (reflexive monitoring). However, they had not reached a consensus of opinion on who performed the DPP (cognitive participation) and how it was performed (collective action).

### Coherence: what the DPP was

RNs, DNs and HCOs had the same view of what the process was and how it was structured. They collectively saw its value in securing the patient’s transition of care from home to the hospital and back home. Even though they agreed about its usefulness and purpose, they had different views about how to interpret the regulations for the DPP. The patients’ had to apply for community service while healthcare was offered to them. RNs, DNs and HCOs thought it was difficult to know the border between the community regulations and the healthcare regulations, and how they could be combined. Meeting together over organizational boundaries and discuss the DPP opened up for a collective view of the process.

### Cognitive participation: Who performs the DPP

RNs, DNs and HCOs expressed that the quality of the DPP improved with specific discharge planners at the hospital wards. They saw it as a legitimate part of their daily work, were engaged with the work tasks and became experts in the area of DP. This led to better continuity and safer timely exchanges of information. In wards where all RNs performed the DPP, there was a risk that everyone’s responsibility became no one’s responsibility. RNs saw the DPP as an extra work task and had to prioritize other work tasks such as patients’ medical treatments, nursing interventions and rounds. RNs expressed that they felt pressure from physicians, DNs and HCOs to be responsible for all coordination and information exchange; meanwhile, RNs wanted them to step up and asked for higher engagement and better collaboration.

DNs and HCOs described that personnel who were confident in their role were better able to handle others’ expectations and had a better understanding of when, what, how and with whom information should be exchanged. They observed that confidence came with work experience and that newly qualified RNs lacked knowledge about DPP and the electronic information system which led to poor information exchange between care providers. RNs expressed that they did not receive any support from physicians, even though physicians were formally responsible for the DPP. Instead, physicians complained that DPs were time-consuming and that the RNs should expedite discharges. DNs expressed that physicians did not prioritize work with the DPP, leading to failure to provide an updated list of medications and to miss prescriptions at patients discharge.

### Collective action: How DPP is performed

RNs, DNs and HCOs experienced different factors that promoted or inhibited work with the DPP. They described that information exchange during the DPP depended on the nurses’ individual skills, beliefs and knowledge. A lack of knowledge impeded the DPP. There was a perceived difference between information provided by the DNs and received by the RNs at patient’s admission and at the DPC. There was a better understanding between the RNs and the DNs for information perceived exchanged at the DPC, and at discharge. Overall, there was a perceived difference between information provided and received electronically, whereas verbally exchanged information was described to work much better.

DNs and HCOs had difficulties preparing for the DPC due to a lack of information in the requests for meetings. RNs often sent DP messages several days later, which made it difficult for DNs and HCOs to participate as expected in the DPP. DNs failed to attend at the DPC due to other demands, workload, and insufficient time for physical meetings. A lack of available and suitable rooms to hold the DPC in at the hospital ward was also an aggravating circumstance for timely DP. All three personnel categories described that HCOs documented their part in the electronic discharge plan directly after the DPC, but DNs did not document their part on time, which could delay the patient’s discharge. RNs had to remind them several times before it was done.

RNs described that patients’ and relatives’ empowerment relied on the information level each RN managed to provide. RNs, DNs and HCOs tried to make the DPP work smoothly for the patients and their relatives but felt that society was not in step with the healthcare system. They stated that increasing elderly population, a non-captive market, the decreasing number of hospital beds, shorter hospital stays, the lower number of sheltered homes and limited personnel resources led to higher demands and a greater workload for all staff working with the DP and impeded the process.

### Reflexive monitoring: How they evaluated the DPP

The RNs, DNs and HCOs evaluation of the DPP showed they had similar views of factors that promoted or inhibited the process, and how to further improve the work. The results showed that RNs, DNs and HCOs thought that double documentation of the DPP in different electronic systems was problematic and time-consuming. It could cause serious breakdowns in the exchange of information and work tasks could therefore be missed. The staff suggested linked systems or a single sign-on to secure the information flow. The results also showed that it was difficult to plan for DPCs that suited everyone’s schedule, which led to many changes and telephone calls. According to RNs, DPC planning had improved because they started to use the electronic schedule consisting of a shared Microsoft Excel spreadsheet. This spreadsheet reduced meeting changes to nearly none, which saved time, but RNs believed that broad implementation countywide was needed. The mapped development requirements showed that the staff thought an electronic calendar, integrated with the electronic information system, would be ideal for the coordination of resources to the DPCs.

RNs, DNs and HCOs thought that DPCs worked best when they all could meet in the same room, but a lack of available time made this difficult to achieve. DNs suggested teleconferences, and HCOs suggested videoconferences as suitable complements to physical meetings. Videoconference meetings would save significant travel time for the DNs and increase chances for a timely DP.

The results showed that DNs and HCOs did not practice a collective follow-up of the patient after discharge. RNs thought it was difficult to know the reasons for patients’ re-admission due to the absence of information about the follow-up in the information system. They thought that development of and access to collective follow-up documentation would enhance the quality of the DPP and save time for the staff involved.

## Discussion

The result of the NPT analysis showed that RNs, DNs and HCOs had reached a consensus of opinion on what the process was (coherence) and how they evaluated the process (reflexive monitoring). However, they had not reached a consensus of opinion of who performed the DPP (cognitive participation) and how it was performed (collective action). This could be interpreted as that they agreed on the overall DPP structure but experienced differences in the work culture. The culture can be defined as a deep structure that finds expression in people’s learned, shared, and inherited knowledge, beliefs, convictions, morals and laws that guide their way of thinking, decision-making and action [46]. In this study, the implementation of the DPP meant that different work cultures needed to make changes in their daily practice to secure patients’ transfer from home to the hospital and back home. The results showed there was limited understanding of each other’s roles and workload and a lack of knowledge in the performance of the DPP, resulting in a delayed communication within and between the different organizations, which was an inhibitor for the DPP in daily practice. According to Greenhalgh et al. [47], an organization can be seen as a platform for how knowledge is maintained, shared and embedded in everyday work. The way an organization addresses knowledge influences the progress of implementation. Organizations that encourage the staff to reflect on their everyday work are more successful in regard to the implementation of new innovations. In a study by Hofflander et al. [48], time was discovered to be an important aspect that affected the outcome of the implementation process in healthcare. They found that individuals needed time to prepare, to reflect and to understand the new practice and its advantages, and the ways it in which it was implemented. They needed time to test the new practice in everyday work and time to reflect on how it might interplay with existing routines, work tasks and regulations. In this study, a lack of time to engage with and perform the DP was experienced by RNs, DNs and HCOs as one inhibitor to making the process work smoothly and on time. Instead, RNs prioritized more traditional work tasks, such as ward rounds, medications, and nursing interventions, whereas DNs prioritized home visits, and physicians showed low interest in being involved at all. From these findings of cognitive participation and collective action we can interpret that the DPP still has not become normalized in daily practice after 10 years of implementation. However, the specific discharge coordinators were seen as both competent and engaged in the task by their colleges and management. Their role was clear to everyone, and they had legitimated time for the performance of the DPP. Greenhalgh et al. [47] described staff that are engaged in and motivated toward new practices as change facilitators that promote implementation among colleagues. We can therefore interpret that the establishment of specific discharge coordinators might be a solution for the normalization of the DPP.

The FIA project [37] had an agile work method [38, 39] where the RNs, DNs and HCOs involvement and engagement might have contributed to increased coherence and reflexive monitoring. A criticism could be that the project failed to identify the lack of normalization of the DPP and that the focus therefore landed on communication issues rather than on work culture issues. This led to the development of technologically supportive tools to enhance the information exchange without facing work-culture differences.

By generating working definitions of the NPT core areas that reflected our specific study setting and testing these definitions during the mapping process we tried to avoid bias in the analysis. Even if data was sorted into predetermined core areas there was a risk of overlap between the constructs. To minimize bias we moved back and forth between data and the NPT literature, and discussed doubts between all three authors until consensus was reached. This systematic way of working with the analysis strengthens its credibility [49]. Another limitation that could also been seen as a strength is the role of the first author, who was both an informal project leader and a researcher, and well known among the healthcare staff as a development manager at the county council’s IT-department. It was therefore very important to inform those who were involved about which role the first author held in each occasion to prevent confusion. Nonetheless, the healthcare staff felt confidence and trust in already knowing the person. This enhanced the ability to collect meaningful and trustworthy data [49].

## Conclusion

In this study, the NPT analysis was performed after the project ended. The analysis showed that the staff had reached a consensus of opinion regarding what the process was (coherence) and how they evaluated the process (reflexive monitoring) but had not reached a consensus of opinion regarding who performs the DPP (cognitive participation) and how it is performed (collective action). These findings show the necessity to observe the implementation of old practices to better understand the needs of new ones before developing and implementing new supportive tools within healthcare to reach the aim of development and to accomplish sustainable implementation. The NPT offers a generalizable framework for analysis, which can explain and shape the implementation process of old practices, before further development of new practices or supportive tools.

## Availability of data and materials

The data supporting the findings of this article are stored at the Department of Health Science, Luleå University of Technology repository. In line with the Swedish regulations of access to research data [50] a formal request to access data must be made.

## Abbreviations

DP: discharge planning
DPP: discharge planning process
DPC: discharge planning conference
ICT: information and communication technology
EHR: electronic health record
NPT: Normalization Process Theory
RN: registered nurse
DN: district nurse
HCO: homecare organizer
